# Persistence with mirabegron or antimuscarinic treatment for overactive bladder syndrome: Findings from the PERSPECTIVE registry study

**DOI:** 10.1111/luts.12382

**Published:** 2021-05-14

**Authors:** Kevin V. Carlson, Eric S. Rovner, Kavita V. Nair, Anna S. Deal, Rita M. Kristy, John C. Hairston

**Affiliations:** ^1^ Section of Urology, Department of Surgery University of Calgary Calgary Alberta Canada; ^2^ Department of Urology Medical University of South Carolina Charleston South Carolina USA; ^3^ Center for Pharmaceutical Outcomes Research, Department of Clinical Pharmacy University of Colorado, Anschutz Medical Campus, Skaggs School of Pharmacy and Pharmaceutical Sciences Aurora Colorado USA; ^4^ IPC/TeamHealth Johnson City Tennessee USA; ^5^ Astellas Pharma Global Development, Inc. Northbrook Illinois USA

**Keywords:** antimuscarinics, mirabegron, overactive bladder, persistence, PERSPECTIVE study

## Abstract

**Objectives:**

This analysis from the PERSPECTIVE (a Prospective, Non‐interventional Registry Study of Patients Initiating a Course of Drug Therapy for Overactive Bladder) study evaluated treatment persistence with mirabegron or antimuscarinics over a 12‐month period.

**Methods:**

Participants were adults diagnosed with overactive bladder (OAB) by their health care provider (HCP), who were initiating mirabegron or antimuscarinic treatment. The HCP made all treatment decisions, and patients were followed for 12 months with no mandatory scheduled visits. Information requests were sent to patients at baseline and months 1, 3, 6, and 12. Patients were nonpersistent if they switched, discontinued, or added OAB medications/therapies to their initial treatment. Reasons for discontinuation and switching patterns were investigated.

**Results:**

Overall, 1514 patients were included (613 mirabegron and 901 antimuscarinic initiators). Persistence rates decreased steadily over time in both groups. A low proportion of patients added or switched medication at each time point. Unadjusted Kaplan‐Meier analysis showed similar persistence rates for both groups. When the data were adjusted for patient characteristics (age, sex, and OAB treatment status), mirabegron initiators had higher persistence rates. No significant differences were noted in unadjusted median time to end of persistence. However, end of treatment persistence by any cause was longer with mirabegron (median: 9.5 vs 6.7 months for antimuscarinics). HCPs stated that the most common reasons for nonpersistence were no symptomatic improvement and side effect aversion.

**Conclusions:**

Treatment persistence was longer for mirabegron compared with antimuscarinic initiators after controlling for patient characteristics. End of treatment persistence by any cause was also longer with mirabegron.

## 1 INTRODUCTION

Overactive bladder (OAB) is a chronic symptom syndrome affecting more than one in ten North American adults, although the condition is particularly common among the elderly.^1^, ^2^, ^3^, ^4^ Serious ramifications on the quality of life (QoL) and daily living of affected patients have been noted,^5^, ^6^ and the treatment of OAB is estimated to cost over $100 billion in the US each year.^7^

Pharmacotherapy approaches are advocated for OAB symptom management if behavioral and conservative measures do not produce adequate improvement.^8^, ^9^ Owing to their established efficacy and propensity to improve QoL, oral antimuscarinics and the β_3_‐adrenoreceptor agonist, mirabegron, are the recommended principal pharmacologic interventions in North America for patients with OAB symptoms.^9^, ^10^, ^11^, ^12^ Although both types of agent are approved as first‐line therapeutic agents,^9^ patients typically receive antimuscarinics first in clinical practice, and, as such, patients who receive mirabegron tend to be more treatment experienced than patients treated with antimuscarinics.^13^, ^14^, ^15^ However, the clinical utility of antimuscarinic agents is limited by the occurrence of certain adverse events (AEs), including dry mouth, constipation, and urinary retention.^16^ Owing to its different mechanism of action, mirabegron is associated with a lower frequency of these AEs and is thought to have a more favorable safety profile than antimuscarinics.^16^, ^17^, ^18^, ^19^ AEs that have been commonly reported in mirabegron trials include hypertension, nasopharyngitis, and headache, although similar frequencies of these events were also observed in the placebo groups.^20^, ^21^, ^22^

Clinical investigations have shown that patients who continue to persist with OAB medication typically experience significantly improved urinary symptoms and QoL.^12^, ^23^, ^24^ However, despite the objective and patient‐reported benefits associated with pharmacological agents as treatment modalities for OAB symptoms, persistence with these medications consistently decreases over time.^14^, ^15^, ^25^ Furthermore, a retrospective pharmacy claims analysis found that oral antimuscarinics specifically exhibit relatively poor adherence and persistence compared with medications used for other common chronic conditions.^26^ Patient‐reported reasons for discontinuing antimuscarinic use include the medication not working as expected or because of the occurrence of side effects.^27^ In fact, a UK prescription data analysis found that between 65% and 86% of patients discontinue therapy within 1 year depending on the antimuscarinic prescribed.^28^ However, real‐world data collected from retrospective administrative claims databases and observational studies suggest that treatment persistence with mirabegron may be greater than for antimuscarinics in patients with OAB.^15^, ^25^, ^29^

PERSPECTIVE (a Prospective, Non‐interventional Registry Study of Patients Initiating a Course of Drug Therapy for Overactive Bladder) is the first large, prospective, non‐interventional study that involves patients with OAB being prescribed mirabegron or antimuscarinics in North American clinical practice.^30^ The primary analysis from the PERSPECTIVE registry found that there were no differences between mirabegron and antimuscarinics in terms of patient‐reported OAB symptom bother and health‐related quality of life (HRQoL) and investigated the safety of both types of therapeutic.^31^ As persistence with different types of OAB pharmacotherapies is not well examined in prospective clinical practice, this secondary analysis of PERSPECTIVE was conducted to evaluate treatment persistence with mirabegron or antimuscarinics over the 12‐month study period.

## 2 METHODS

### 2.1 Study design

The overall methods used in this study have been reported previously.^30^ Briefly, PERSPECTIVE was conducted in health care centers and sites throughout the US and Canada, and the study population comprised adult patients diagnosed with OAB by their health care provider (HCP). Eligible patients had to be initiating a new course of treatment with either mirabegron or an antimuscarinic. Both treatment‐naïve and treatment‐experienced patients were enrolled, and patients were judged to be treatment experienced if they had received ≥1 OAB medication in the previous 12 months.

The study was conducted in accordance with the Declaration of Helsinki, the protocol was approved by the institutional review board or independent ethics committee at each site, and all patients provided informed consent.

### 2.2 Study assessments

All clinical assessments were performed during a routine health care appointment, and all decisions regarding treatment were made at the discretion of the treating HCP in accordance with their usual practices. Patients were followed for up to 12 months, with no mandatory scheduled visits after enrollment. Demographic data were collected from patients at baseline.

Using patient‐reported data, this analysis was conducted to compare persistence with the first medication the patients received (ie, initial mirabegron or antimuscarinic treatment) over a 12‐month period. Requests for information were sent by email link to patients at baseline (days 0‐7), month 1 (days 30‐45), month 3 (days 91‐125), month 6 (days 182‐208), and month 12 (days 336‐377) to be completed electronically in their preferred language (English, French, or Spanish). Exposure data that were collected from patients included medication name, date started, date stopped, and reason for discontinuation. Along with these parameters, HCPs were also asked to provide details on medication dose, dosage form (eg, instant release, extended release, or transdermal), details of any dose titration, and number of doses per day. For this analysis, a patient was classified as nonpersistent with their initial treatment if the patient switched OAB medication, discontinued medication, or added other OAB medications or OAB therapies including onabotulinumtoxin A or sacral neuromodulation (regardless of the reason for the alteration in medication). These patients were considered nonpersistent for the remainder of the study. However, dose alterations were not considered to be equivalent to the initiation of a new treatment, and therefore patients were classified as persisting with treatment if the dose of their medication had been altered. Reasons for discontinuation and switching patterns were also explored.

### 2.3 Statistical analyses

Statistical data were analyzed with SAS (version 9.2 or higher; SAS, Cary, North Carolina). Patients were not excluded from analysis if data were missing for a particular time point. Therefore, variations were possible in the number of patients analyzed over time. Antimuscarinic data were assessed in terms of the group of therapeutics as a whole rather than each individual medication.

Unadjusted persistence rates with initial OAB treatment were assessed in terms of the proportion of patients at each follow‐up time point. The persistence rate was defined as:

(number of patients on initial OAB treatment at each follow‐up time point / total number of patients with available data at each time point) × 100.

Adding or switching medication was categorized according to the initial OAB treatment and the addition or switch to a second‐line OAB medication type. This included adding/switching to mirabegron, adding/switching to antimuscarinics, discontinuation of initial treatment, or switching to onabotulinumtoxin A or sacral neuromodulation. Rates of switching, adding on, or discontinuing were calculated as:

(number of patients switching/adding on/discontinuing initial OAB treatment at each follow‐up time point / total number of patients with available data at each time point) × 100.

The Kaplan‐Meier method was used to estimate persistence with initial treatment and was adjusted for baseline age, sex, and OAB treatment status (naïve vs experienced). The log‐rank test was used to compare unadjusted median time to end of persistence between mirabegron and antimuscarinic initiators. Analyses were conducted for all patients and according to age group, sex, and OAB treatment status. A cumulative incidence function for end of treatment persistence by any cause was estimated according to the initial treatment group using a competing risks regression (Fine‐Gray) model with effects for age, sex, and OAB treatment status. The same methodology was used to evaluate the individual components that contributed to the persistence by any cause parameter, namely switching treatment, adding on treatment, or stopping all treatment. Hazard ratios for the association between persistence and treatment group were calculated and adjusted for baseline age, sex, and OAB treatment status prior to baseline. Reasons for discontinuation, switching, or adding on treatment as reported by patients (verbatim text) and HCPs were summarized in terms of number and percentage of patients for each specific medication. The chi‐square test was used to evaluate the overall reasons for discontinuation as reported by the HCPs. *P* values of less than .05 were judged to be nominally significant without multiplicity adjustment.

## 3 RESULTS

### 3.1 Study population

The study population data have been reported previously.^31^ In brief, a total of 1514 patients were included in PERSPECTIVE (613 mirabegron initiators and 901 antimuscarinic initiators). The most frequently initiated antimuscarinics were oxybutynin (423 [46.9%] patients), solifenacin (298 [33.1%] patients), and tolterodine (90 [10.0%] patients). The occurrence of a patient‐support program in Canada, apart from British Columbia and Quebec, meant that up to 100% of the cost for mirabegron was covered for some patients during this study. This meant that a greater proportion of the patients from Canada initiated mirabegron treatment (223 [70.6%] patients) compared with their US counterparts (390 [32.6%] patients). Overall, most of the patients were female (1113 [73.5%] patients) with a mean age of 62.2 ± 14.4 years. Compared with the mirabegron initiators, a lower proportion of the antimuscarinic initiators were male (189 [30.8%] patients vs 212 [23.5%] patients, respectively) and treatment experienced (178 [29.0%] patients vs 169 [18.8%] patients, respectively). Patient age did not appear to have an effect on the treatment that was initiated (≥60 years old—mirabegron initiators: 390 [63.6%] patients, antimuscarinic initiators: 549 [60.9%] patients). However, treatment‐experienced patients did appear to be older than treatment‐naïve patients (≥60 years old—treatment experienced: 237/347 [68.3%] patients, treatment naïve: 702/1167 [60.2%] patients). In terms of medications that are used to treat benign prostatic hyperplasia, 95 (6.3%) and 41 (2.7%) patients were receiving concomitant treatment at baseline with α_1_‐adrenoreceptor antagonists and 5α‐reductase inhibitors, respectively (Table S1).

### 3.2 Persistence

The unadjusted persistence rates decreased steadily in both the mirabegron and antimuscarinic groups (Table 1). Among the mirabegron initiators, approximately 1.2% of patients switched to antimuscarinics at each time point (except for 0.3% at month 6) and between 1.3% and 3.3% of patients added an antimuscarinic to their treatment regimen. For the antimuscarinic initiators, ≤1.7% of patients switched to mirabegron or another antimuscarinic during the follow‐up period. Between 0.2% and 1.4% of the antimuscarinic initiators added mirabegron, while between 0.5% and 1.8% of the antimuscarinic initiators added another antimuscarinic. No statistically significant differences were observed between treatment groups in terms of the patterns of discontinuation according to whether a patient added or switched medication (Table S2).

**TABLE 1:** Prevalence of specific adding/switching patterns at follow‐up visit time points as reported by patients

Adding/switching pattern		Follow‐up visit time point			
Initial medication	Outcome	Month 1 (n = 1013)	Month 3 (n = 1057)	Month 6 (n = 854)	Month 12^a^ (n = 859)
Mirabegron^b^	No. of patients (n)	376	392	303	311
	Persisted with initial treatment	323 (85.9)	311 (79.3)	217 (71.6)	206 (66.2)
	Switched to an antimuscarinic	5 (1.3)	4 (1.0)	1 (0.3)	4 (1.3)
	Added an antimuscarinic	12 (3.2)	9 (2.3)	10 (3.3)	4 (1.3)
	Discontinued initial treatment	18 (4.8)	55 (14.0)	61 (20.1)	80 (25.7)
	Switched to onabotulinumtoxin A	0	1 (0.3)	0	0
	Switched to sacral neuromodulation	0	0	0	0
	Missing all treatment information	8 (2.1)	8 (2.0)	12 (4.0)	18 (5.8)
Antimuscarinics^b^	No. of patients (n)	637	665	551	548
	Persisted with initial treatment	566 (88.9)	532 (80.0)	413 (75.0)	376 (68.6)
	Switched to mirabegron	1 (0.2)	7 (1.1)	3 (0.5)	0
	Switched to another antimuscarinic	11 (1.7)	5 (0.8)	2 (0.4)	2 (0.4)
	Added mirabegron	9 (1.4)	8 (1.2)	2 (0.4)	1 (0.2)
	Added another antimuscarinic	9 (1.4)	12 (1.8)	7 (1.3)	3 (0.5)
	Discontinued initial treatment	42 (6.6)	112 (16.8)	110 (20.0)	141 (25.7)
	Switched to onabotulinumtoxin A	0	1 (0.2)	0	2 (0.4)
	Switched to sacral neuromodulation	0	0	0	0
	Missing all treatment information	8 (1.3)	19 (2.9)	18 (3.3)	18 (3.3)

*Note*: Data are given as n (%), unless otherwise stated.

^a^ The month‐12 time point includes data that were acquired in the wider window including >12 months into the follow‐up period.

^b^ For each time point, some patients may have missing data regarding second‐line medication, while other patients may contribute to ≥2 second‐line medication categories. Therefore, the percentages may not add up to 100%.

**FIGURE 1:**
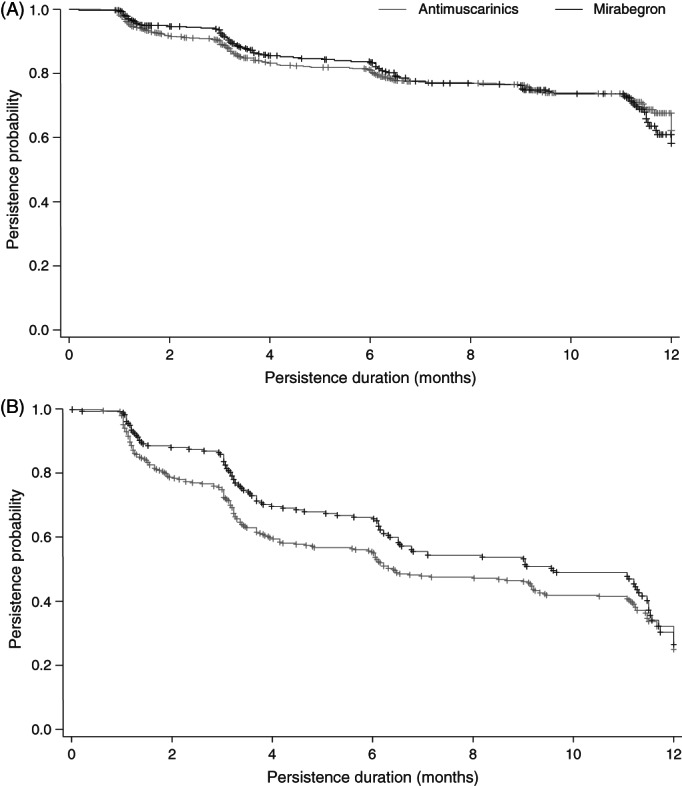
Unadjusted (A) and adjusted (B) Kaplan‐Meier curves of treatment persistence with mirabegron and antimuscarinics. Panel B has been adjusted for baseline age, sex, and prior overactive bladder medication use

The unadjusted Kaplan‐Meier analysis showed that patients initiating mirabegron or antimuscarinics had similar rates of persistence during follow‐up (Figure 1A). However, when the data were adjusted for baseline age, sex, and prior OAB medication use, the persistence curves for initial mirabegron or antimuscarinic treatment separated by month 2 of the study, with mirabegron initiators showing higher rates of persistence (Figure 1B).

Although low numbers of patients were included, no statistically significant differences were noted between the mirabegron and antimuscarinic initiators in terms of unadjusted median time to end of persistence for all patients and for each of the subgroup analyses (Table 1, 2). In terms of the age group analysis, the longest median persistence results were observed for the 18 to <30 years age group (mirabegron initiators: 10.3 months) and the 40 to <50 years age group (antimuscarinic initiators: 10.7 months). For both groups, female patients persisted with treatment longer than male patients, and treatment‐naïve patients persisted longer than patients who were treatment experienced.

**TABLE 2:** Comparison of persistence with mirabegron and antimuscarinics in terms of all patients and according to baseline age, sex, and OAB treatment status

Group	Mirabegron (n = 613)			Antimuscarinics (n = 901)			*P* value^b^
	n	No. of patients persisting (n)^a^	Median time to end of persistence in months	n	No. of patients persisting (n)^a^	Median time to end of persistence in months	
Total	613	21	9.0	901	23	9.2	.8829
Age group in years							.9616
≥18 to <30	20	1	10.3	25	0	3.2	
≥30 to <40	28	1	9.4	42	0	5.1	
≥40 to <50	64	1	6.5	112	1	10.7	
≥50 to <60	111	5	9.6	173	2	9.6	
≥60	390	13	9.0	549	20	9.3	
Sex							.9632
Male	189	1	6.2	212	7	6.5	
Female	424	20	9.4	689	16	9.3	
OAB treatment status							.2414
Experienced	178	3	6.8	169	1	4.8	
Naïve	435	18	9.1	732	22	9.7	

Abbreviation: OAB, overactive bladder.

^a^ Patient numbers were derived using a continuous time analysis, and the data reflect the patients who had observations that occurred on or after month 12.

^b^ *P* values calculated from separate log‐rank tests for baseline age, sex, and OAB treatment status.

**FIGURE 2:**
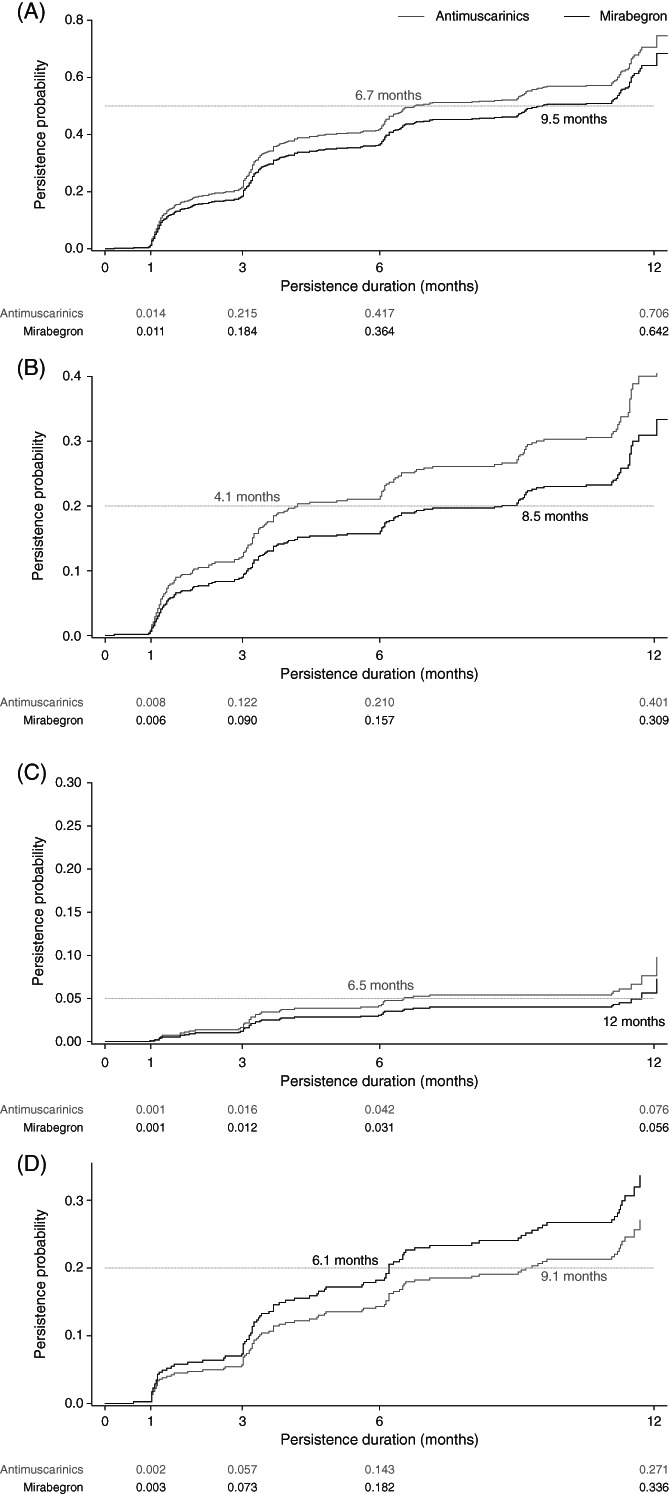
Cumulative end of treatment persistence with mirabegron and antimuscarinics according to any cause (A) or due to switching treatment (B), adding on treatment (C), or stopping all treatment (D). The gray dashed line represents an estimated probability midpoint for each treatment group

Analysis of the end of treatment persistence by any cause data showed that clear separation was observed in favor of mirabegron (median time to end of treatment persistence: 9.5 months vs 6.7 months for antimuscarinics; Figure 2A). This observation may be due to antimuscarinic initiators switching treatment (median time: 4.1 months vs 8.5 months for mirabegron; Figure 2B) or adding a treatment (median time: 6.5 months vs 12 months for mirabegron, Figure 2C) sooner than mirabegron initiators. In contrast, mirabegron initiators who stopped all treatment during follow‐up did so sooner than antimuscarinic initiators (median time: 6.1 months vs 9.1 months, respectively; Figure 2D).

**FIGURE 3:**
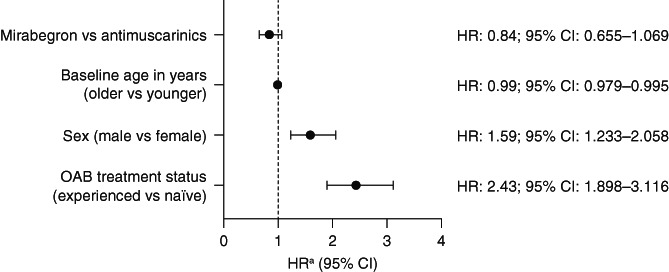
Association between end of persistence and treatment groups based on a covariate‐adjusted Cox proportional hazard model. CI, confidence interval; HR, hazard ratio; OAB, overactive bladder. ^a^Independent variables used in the model included treatment group, baseline age, sex, and OAB treatment status

Data from the adjusted Cox proportional hazard model also showed that initial treatment had no effect on time to end of persistence (Figure 3). However, older, female, and treatment‐naïve patients were statistically more likely to persist with treatment for a longer period of time than their younger, male, and treatment‐experienced counterparts.

Data on the reasons for discontinuing, switching, or adding on a treatment were available for 213 (34.7%) mirabegron initiators and 200 (22.2%) antimuscarinic initiators. According to the HCPs involved in the study, the biggest reasons for patients discontinuing, switching, or adding on treatment were no improvement in symptoms and aversion to the side effects (Table 3). The proportion of patients who discontinued due to no improvement in symptoms was slightly higher in the mirabegron group (101 [47.4%] mirabegron initiators, 85 [42.5%] antimuscarinic initiators), whereas the proportion of patients who discontinued due to side effects was higher in the antimuscarinic group (48 [22.5%] mirabegron initiators, 58 [29.0%] antimuscarinic initiators). The chi‐square test revealed that there was no overall statistically significant difference between groups in the reasons for discontinuation (*χ* ^2^ = 0.9059). In terms of the verbatim patient‐reported data, common reasons for not persisting with the initial treatment were the treatment did not work, cost, and side effects. The side effects reported included blurred vision, headaches, constipation, dry mouth, and nausea.

**TABLE 3:** Patient reasons for discontinuing, switching, or adding on treatment as reported by the HCPs

Reason	Mirabegron (n = 613)	Antimuscarinics (n = 901)
No. of patients (n)	213	200
Did not complete prescription	1 (0.5)	0
Did not improve symptoms	101 (47.4)	85 (42.5)
Did not like side effects	48 (22.5)	58 (29.0)
No insurance cover	20 (9.4)	17 (8.5)
Other	43 (20.2)	40 (20.0)

*Note*: Data are given as n (%), unless otherwise stated.

Abbreviation: HCP, health care provider.

## 4 DISCUSSION

In this secondary analysis from the PERSPECTIVE registry, patients with OAB initiating a new course of mirabegron persisted with treatment longer than those initiating antimuscarinics after controlling for differences in patient characteristics between groups. End of treatment persistence by any cause was also longer with mirabegron than with antimuscarinic treatment. It is important to note, however, that no statistical analyses were conducted on these specific results. Furthermore, no additional notable differences in persistence were observed between the two groups.

In agreement with the specific persistence findings mentioned above, previous real‐world analyses conducted in Canada, Japan, Spain, the US, and the UK typically showed that patients treated with mirabegron remained on treatment longer than those treated with antimuscarinics.^13^, ^14^, ^15^, ^32^, ^33^, ^34^ In these previous studies, 12‐month persistence rates of between 14% and 38% were noted for mirabegron, and rates of between 3% and 25% were reported with antimuscarinics. Conversely, the opposite finding was noted in a Japanese urology clinic study, which reported 12‐month persistence rates of 12.2% and 20.1% with mirabegron and solifenacin, respectively.^35^ It is important to note, however, that the Japanese urology clinic study was a prospective, randomized trial when the other studies mentioned above were retrospective database analyses, and these differences in study design may partially explain the contradictory results obtained.^13^, ^14^, ^15^, ^32^, ^33^, ^34^, ^35^ In addition, the differences observed in the Japanese study were not statistically significant, which was possibly due to the relatively small study population of 148 patients.^35^ The persistence rates reported in the above studies are notably lower than the present study, where approximately two‐thirds of patients were persisting with mirabegron or antimuscarinics at month 12. The differences observed may be due to the unique nature of the PERSPECTIVE study compared with the other investigations. Owing to the prospective nature of the study, the patients enrolled in PERSPECTIVE may have been more likely to respond to the requests for information if they were still actively taking the medication. Furthermore, the results were derived using the number of patients who responded to the email as the patient population rather than an intent‐to‐treat population, which would have included a higher total number of patients.

This investigation showed that treatment persistence was longer with mirabegron when the Kaplan‐Meier results were adjusted for baseline age, sex, and prior OAB medication use. However, no difference was noted in the unadjusted results. These findings are potentially due to the fact that mirabegron initiators were more likely to be male and treatment experienced than their antimuscarinic counterparts. In agreement with these findings, previous persistence studies typically showed that higher proportions of males and treatment‐experienced patients were noted in mirabegron compared with antimuscarinic cohorts.^13^, ^14^, ^15^, ^25^ In this study, the age of the patients did not appear to have an effect on the treatment that was initiated. Furthermore, the fact that a greater proportion of mirabegron initiators were treatment experienced may partially explain why end of treatment persistence by any cause was longer in this group than in the antimuscarinic initiators group. The patients who initiated mirabegron may have therefore been more familiar with the efficacy of OAB medication and the potential adverse effects associated with treatment.

No statistically significant differences in the persistence results were observed in PERSPECTIVE. This finding was potentially due to the study design as there was no requirement for patients to attend scheduled visits during the follow‐up period. The lack of statistically significant differences in persistence may explain why similar patient‐reported OAB symptom bother and HRQoL results were observed for both treatments in the present study.^31^

In our investigation, older, female, and treatment‐naïve patients were more likely to persist with treatment for a longer period of time. The finding that older patients persisted with OAB therapy longer than younger patients has been previously reported in several persistence studies involving mirabegron and antimuscarinics.^13^, ^15^, ^25^ However, a further study found no relationship between patient age and OAB treatment persistence,^14^ and a UK‐based hospital prescription analysis found that younger age was associated with improved persistence with mirabegron therapy.^36^ Furthermore, previous investigations support the results of this study in so much as female patients were found to persist with OAB medication for a longer period of time than their male counterparts.^13^, ^37^ Conversely, a UK clinical practice study found no difference in persistence rates between the sexes,^14^ and a Japanese real‐world analysis study found that males typically persisted with mirabegron and antimuscarinic therapy longer than females.^15^ In contrast to the present study, previous persistence studies have typically found that treatment‐experienced patients persist with mirabegron and antimuscarinic therapy longer than treatment‐naïve patients.^13^, ^14^, ^15^, ^25^ The discrepancies between our findings and the results of previous investigations may be partially due to the design of the studies. The previous studies were database analyses, while the present study was a clinical practice investigation. In support of this hypothesis, a retrospective, real‐life study found that treatment‐naïve patients persisted with mirabegron treatment longer than patients who had received previous anticholinergic therapy.^38^

The switching analyses showed that a low percentage of patients switched treatments during the present study. The highest percentage of patients who switched treatment at any given time point was 1.7% for the antimuscarinic initiators who switched to another antimuscarinic at the month‐1 time point. Compared with the present study, higher rates of switching have been typically observed in previous studies investigating persistence with mirabegron and antimuscarinics, although varied results have been obtained. For example, in a large retrospective US database analysis, a total of 14% and 4% of the patients who received treatment with mirabegron or an anticholinergic, respectively, switched treatment during a 12‐month follow‐up period.^33^ In addition, 28% and 60% of patients switched to another drug following initial treatment with mirabegron or antimuscarinics in a small 8‐week Japanese prospective study.^39^ Similar findings were observed in a 48‐month prospective study, which found that 60.8% of patients switched from solifenacin to mirabegron therapy.^40^

According to the HCPs, the most prominent reasons for patients discontinuing treatment were no improvement in symptoms and aversion to the side effects. Although only a few studies have examined the reasons for discontinuing OAB treatment, our findings are consistent with the results of previous persistence studies. The results of a phase 1 screening survey showed that the most common reasons for discontinuing OAB medication were the medication not working as expected, switching to a new medication, learning to get by without medication, and experiencing side effects.^41^ Furthermore, a Japanese single‐center retrospective study found that the biggest reasons for discontinuing mirabegron treatment were unmet treatment expectations, the occurrence of AEs, and symptom improvement.^42^ In our study, the proportion of patients who discontinued following no improvement in their symptoms was slightly higher in the mirabegron group, whereas the proportion of patients who discontinued due to side effects was higher in the antimuscarinics group. The latter finding is potentially due to the higher incidence of anticholinergic side effects that are noted with antimuscarinics compared with mirabegron.^16^

Despite the promising results obtained in the present study, long‐term treatment of a chronic disease such as OAB represents a challenge for patients in terms of adhering to and persisting with prescribed medication. The decline in patient persistence in both treatment groups over time emphasizes the importance of using strategies that improve patient treatment experiences. For example, patient‐support programs have been shown to improve persistence with and/or adherence to medications for the treatment of other chronic diseases.^43^, ^44^, ^45^ Indeed, an initial study found that patients with OAB who followed a patient‐support plan found that it was informative and feasible to implement and that they were satisfied with several aspects of the plan.^46^ The widespread use of support plans could therefore prove beneficial in this setting to improve treatment persistence.

Owing to the varied population involved, we believe that this study more accurately reflects the real‐world situation compared with a clinical trial population, where specific inclusion and exclusion criteria have to be satisfied for enrollment. In addition, this investigation was a prospective study and therefore does not suffer from the potential data deficiencies that are inherent with retrospective database investigations. However, this study does have some limitations. As specific scheduled follow‐up visits were not included as part of the study protocol, the quantity of persistence data available throughout the study varied according to the follow‐up time point. Furthermore, data on the reasons for discontinuing, switching, or adding on a treatment were only available for a minority of patients. Additionally, no data were captured on whether the patients actually took the medication. Therefore, patients could have been identified as persisting with treatment when they were not taking the medication as prescribed by their HCP. Lastly, if a patient diary had been used during this study, it may have increased patient awareness about the current status of their condition and may have therefore led to increased persistence with the study medication.

In conclusion, the present study is the first observational study conducted in North America to investigate patient persistence with OAB medication following treatment with either mirabegron or antimuscarinics. Specific results from this study showed that patients who received mirabegron persisted with treatment for a longer period of time than the patients who initiated antimuscarinics, although no statistical differences were observed. Regardless of the treatment used, approximately two‐thirds of patients were still persisting with their initial treatment after 12 months. These high rates of persistence could lead to symptomatic improvements in patients with OAB.

## DISCLOSURE

Kevin V. Carlson, Eric S. Rovner, Kavita V. Nair, and Anna S. Deal are members of the PERSPECTIVE Scientific Advisory Committee that receives compensation from Astellas. Rita M. Kristy and John C. Hairston are employees of Astellas Pharma Global Development, Medical Affairs.

## Supporting information


**TABLE S1.** Concomitant α_1_‐adrenoreceptor antagonist and 5α‐reductase inhibitor medication use at baseline

**TABLE S2.** Prevalence of switching OAB therapy or adding‐on OAB therapy at follow‐up visit time points as reported by patients

## Data Availability

Researchers may request access to anonymized participant‐level data, trial‐level data, and protocols from Astellas‐sponsored clinical trials at www.clinicalstudydatarequest.com. For the Astellas criteria on data sharing, see: https://clinicalstudydatarequest.com/Study-Sponsors/Study-Sponsors-Astellas.aspx.
