# Supplementary material for: Persistence with mirabegron or antimuscarinic treatment for overactive bladder syndrome: Findings from the PERSPECTIVE registry study
Source: Low Urin Tract Symptoms. 2021 May 14;13(4):425–34. doi: 10.1111/luts.12382 (PMC8518921; doi:10.1111/luts.12382)
Supplement: Supplementary file 1 — TABLE S1. Concomitant α1‐adrenoreceptor antagonist and 5α‐reductase inhibitor medication use at baseline [file LUTS-13-425-s001.docx]

TABLE S1 Concomitant α_1_-adrenoreceptor antagonist and 5α-reductase inhibitor medication use at baseline

| **Medication** | **Mirabegron (n = 613)** | **Antimuscarinics (n = 901)** | **Total (n = 1514)** |
| --- | --- | --- | --- |
| α_1_-adrenoreceptor antagonists | 54 (8.8) | 41 (4.6) | 95 (6.3) |
| Tamsulosin | 19 (3.1) | 21 (2.3) | 40 (2.6) |
| Tamsulosin hydrochloride | 11 (1.8) | 11 (1.2) | 22 (1.5) |
| Alfuzosin | 11 (1.8) | 2 (0.2) | 13 (0.9) |
| Doxazosin | 4 (0.7) | 4 (0.4) | 8 (0.5) |
| Silodosin | 4 (0.7) | 3 (0.3) | 7 (0.5) |
| Terazosin | 3 (0.5) | 0 | 3 (0.2) |
| Alfuzosin hydrochloride | 1 (0.2) | 1 (0.1) | 2 (0.1) |
| Dutas-t | 1 (0.2) | 0 | 1 (0.1) |
| Prazosin hydrochloride | 0 | 1 (0.1) | 1 (0.1) |
| 5α-reductase inhibitors | 24 (3.9) | 17 (1.9) | 41 (2.7) |
| Finasteride | 16 (2.6) | 9 (1.0) | 25 (1.7) |
| Dutasteride | 8 (1.3) | 8 (0.9) | 16 (1.1) |

*Note*: Data are given as n (%). The medication categories are listed according to the term used by the reporting physician.
