# Supplementary material for: Persistence with mirabegron or antimuscarinic treatment for overactive bladder syndrome: Findings from the PERSPECTIVE registry study
Source: Low Urin Tract Symptoms. 2021 May 14;13(4):425–34. doi: 10.1111/luts.12382 (PMC8518921; doi:10.1111/luts.12382)
Supplement: Supplementary file 2 — TABLE S2. Prevalence of switching OAB therapy or adding‐on OAB therapy at follow‐up visit time points as reported by patients [file LUTS-13-425-s002.docx]

TABLE S2 Prevalence of switching OAB therapy or adding on OAB therapy at follow-up visit time points as reported by patients

|  | **Mirabegron (n = 613)** | | | | | **Antimuscarinics (n = 901)** | | | | | ***P* value^b^** |
| --- | --- | --- | --- | --- | --- | --- | --- | --- | --- | --- | --- |
|  | **Month 1 (n = 376)** | **Month 3 (n = 392)** | **Month 6 (n = 303)** | **Month 12^a^ (n = 311)** | **Total (n)** | **Month 1 (n = 637)** | **Month 3 (n = 665)** | **Month 6 (n = 551)** | **Month 12^a^ (n = 548)** | **Total (n)** |  |
| Patients persistent with initial treatment | 323 (85.9) | 311 (79.3) | 217 (71.6) | 206 (66.2) | 505 | 566 (88.9) | 532 (80.0) | 413 (75.0) | 376 (68.6) | 718 | .1919 |
| Patients who discontinued initial treatment^c^ | 18 (4.8) | 55 (14.0) | 61 (20.1) | 80 (25.7) | 108 | 42 (6.6) | 112 (16.8) | 110 (20.0) | 141 (25.7) | 183 |  |
| Any adding/switching medication | | | | |  |  |  |  |  |  | .4518 |
| Yes^d^ | 17 (4.5) | 13 (3.3) | 11 (3.6) | 8 (2.6) | 49 | 30 (4.7) | 32 (4.8) | 14 (2.5) | 6 (1.1) | 82 |  |
| No | 359 (95.5) | 379 (96.7) | 292 (96.4) | 303 (97.4) | 564 | 607 (95.3) | 633 (95.2) | 537 (97.5) | 542 (98.9) | 819 |  |
| Switching from initial to any other OAB medication | | | | |  |  |  |  |  |  | .1932 |
| Yes^d^ | 5 (1.3) | 4 (1.0) | 1 (0.3) | 4 (1.3) | 14 | 12 (1.9) | 12 (1.8) | 5 (0.9) | 2 (0.4) | 31 |  |
| No | 371 (98.7) | 388 (99.0) | 302 (99.7) | 307 (98.7) | 599 | 625 (98.1) | 653 (98.2) | 546 (99.1) | 546 (99.6) | 870 |  |
| Adding on any other OAB medication to initial treatment | | | | |  |  |  |  |  |  | .9676 |
| Yes^d^ | 12 (3.2) | 9 (2.3) | 10 (3.3) | 4 (1.3) | 35 | 18 (2.8) | 20 (3.0) | 9 (1.6) | 4 (0.7) | 51 |  |
| No | 364 (96.8) | 383 (97.7) | 293 (96.7) | 307 (98.7) | 578 | 619 (97.2) | 645 (97.0) | 542 (98.4) | 544 (99.3) | 850 |  |
| Patient-reported nonpharmacologic intervention | | | | |  |  |  |  |  |  | .1551 |
| Yes^d,e^ | 9 (2.4) | 5 (1.3) | 6 (2.0) | 8 (2.6) | 26 | 7 (1.1) | 10 (1.5) | 3 (0.5) | 8 (1.5) | 26 |  |
| No | 367 (97.6) | 387 (98.7) | 297 (98.0) | 303 (97.4) | 587 | 630 (98.9) | 655 (98.5) | 548 (99.5) | 540 (98.5) | 875 |  |

*Note*: Data are given as n (%), unless otherwise stated.
Abbreviation: OAB, overactive bladder.
^a^The month-12 time point includes data that were acquired in the wider window including >12 months into the follow-up period.
^b^Calculated using the chi-square test.
^c^Frequencies are cumulative: a patient who discontinued initial treatment during a prior visit window is also counted in the current visit window (if the patient has current visit data) and in “Total”.
^d^Frequencies are not cumulative: a prior event does not count in the current visit window but in “Total” only. Out-of-window events do not count.
^e^A patient may have multiple interventions during one visit window or across visit windows. Within each visit window and within “Total” a patient is counted only once.
